# Data survey on public-private professionals role in building control measures within the Nigerian construction industry

**DOI:** 10.1016/j.dib.2018.08.193

**Published:** 2018-09-05

**Authors:** Rapheal A. Ojelabi, Olabosipo I. Fagbenle, Lekan M. Amusan, Adedeji O. Afolabi, Opeyemi Oyeyipo

**Affiliations:** Department of Building Technology, Covenant University, Nigeria

**Keywords:** Building control measures, Public and private institutions, Sustainable practice, Construction expert

## Abstract

The data survey focused on building control measures which are critical in attaining sustainable built environment. The data examined the public and private professional׳s performance of building control measures and its barriers in Lagos. The targets consist of sixty (60) construction experts in public and private construction firms operating in Lagos. The sample size was generated from the pool of registered construction professional׳s from private and public construction organizations in Lagos using random sampling technique. Descriptive statistics and inferential tests which include Kruskal-Wallis and Mann-Whitney U tests were performed on the dataset generated. The data harvested will avail the construction expert׳s in public and private construction industry on the need for partnership in a bid to enhance building control measures performance in Lagos.

**Specifications table**

**Table:** 

Subject area	Building Construction.
More specific subject area	Building Control.
Type of data	Tables and Figures
How data was acquired	Cross-sectional Survey design
Data format	Raw, analyzed.
Experimental factors	A random sampling of Construction experts
Experimental features	Public-Private Construction Experts Role in Building Control Measures Performance and Barriers Within Nigeria.
Data source location	Lagos, Nigeria.
Data accessibility	*All the data are in this data article*
Related research article	*Please cite the author, title and publication status of the most relevant related research article here, if applicable.*

**Value of the data**

• The data when analyzed compared with existing data on building control can help identify the sustainable ways of achieving sustainable building control measures.
• The dataset can help construction experts to ascertain the state of building control practices in a bid to enhance their performance and preserve the built environment [1], [2], [3], [4], [5], [6].
• The dataset will avail the public and private construction institutions of the need for partnership in order to enhance building control measures and manage the barriers to its effective performance.

## 1 Data

The deficit in housing provisions due to the alarming increase in cities population has led to the prevalence of abuse of the built environment in a bid to meeting the housing challenge [7], [8], [9]. The data explored the role of construction experts in public and private construction organizations performance in building control measures and the barriers militating against its practice in Lagos. A well-structured questionnaire was used to generate the data from sixty (60) construction experts operating in the study area. The data generated from the construction experts are as follow: data on descriptive statistics of the experts which include age, academic qualification, respondents sector, profession and professional membership body is presented in Table 1, data on construction expert׳s perception on performance of building control measures in developing city (Table 2) and data on construction expert׳s perception on barriers to building control measures (Table 3). The analyzed data was also used to generate inferential statistics using Kruskal-Wallis and Mann-Whiney U as presented in Table 4, Table 5, Table 6, Table 7, Table 8, Table 9.

**Table 1:** Descriptive characteristics of respondents.

**Characteristics**	**Frequency**	**Percentage**
**Ages**		
<20 years	6.0	10.0
31–40 years	20.0	33.3
41–50 years	27.0	45.0
51–60 years	7.0	11.7
**Total**	**60.0**	**100**
**Academic qualification**		
HND	11.0	18.3
B.Sc/B.Tech	21.0	35.0
M.sc/MBA	28.0	46.7
**Total**	**60.0**	**100**
**Sector**		
Private	37.0	61.7
Public	23.0	38.3
**Total**	**60.0**	**100**
**Profession**		
Architect	13.0	21.7
Quantity surveyor	9.0	15.0
Builder	24.0	40.0
Civil engineer	8.0	13.3
Urban and regional planner	6.0	10.0
**Total**	**60.0**	**100**
**Professional Body**		
Nigeria Institute of Architect (NIA).	13.0	21.7
Nigeria Society of Engineers (NSE).	8.0	13.3
Nigeria Institute of Building (NIOB).	25.0	41.7
Nigeria Institute of Quantity Surveyors (NIQS).	8.0	13.3
Nigeria Institute of Town Planners (NITP).	6.0	10.0
**Total**	**60.0**	**100**

**Table 2:** Construction expert׳s perspective on performance of building control procedures.

Building Control Procedures	Mean	Remarks
The handling and examining of building plans for approval	4.00	Often
The assessment of the progress of building work.	3.62	Sometimes/Often
Zero tolerance for illegal, hazardous and violations of legal conditions related in building	3.22	Sometimes/Often
The assessment of building completed according to accepted building requirements for giving out certificates of occupancy	3.15	Sometimes/Often
Records keeping in relation to building and related authorization	2.85	Rarely/Sometimes
Creating a legal framework for instantaneous and uninterrupted audit and certification of existing buildings	2.32	Rarely/Sometimes
The synchronization with other legal bodies to meet their requirements.	1.95	Never/Rarely

**Table 3:** Construction experts perception on barriers to building control procedures.

	Mean	Remarks
Bureaucratic process.	4.20	High/Very High
Corruption.	4.17	High/Very High
Greed.	4.10	High/Very High
Lack of coordination by agencies.	4.07	High/Very High
Inadequate building control legal framework.	4.05	High/Very High
The inadequacy of building control specialist	4.03	High/Very High
Lack of training.	4.00	High
Lack of enforcement mechanism.	3.92	Moderate/High
Lack of personnel.	3.77	Moderate/High
Inadequate fund for agencies.	3.65	Moderate/High

**Table 4:** Kruskal Wallis result on construction experts mean rank on building control procedures.

Building Control Procedures	Profession	N	Mean Rank
The handling and examining of building plans for approval.	Architect	13	30.46
	Quantity surveyor	9	37.28
	Builder	24	28.94
	Civil Engineer	8	31.25
	Urban and Regional Planners	6	25.67
	**Total**	**60**	
The assessment of the progress of building work.	Architect	13	32.69
	Quantity surveyor	9	26.39
	Builder	24	32.19
	Civil Engineer	8	31.00
	Urban and Regional Planners	6	24.50
	**Total**	**60**	
The assessment of building completed according to accepted building requirements for giving out certificates of occupancy.	Architect	13	32.12
	Quantity surveyor	9	40.83
	Builder	24	29.00
	Civil Engineer	8	24.06
	Urban and Regional Planners	6	26.08
	Total	60	
Zero tolerance for illegal, hazardous and violation of legal requirements related to the building.	Architect	13	27.31
	Quantity surveyor	9	33.67
	Builder	24	30.88
	Civil Engineer	8	31.38
	Urban and Regional Planners	6	30.00
	**Total**	**60**	
Records keeping in relation to building and related authorization.	Architect	13	28.50
	Quantity surveyor	9	30.78
	Builder	24	29.71
	Civil Engineer	8	36.63
	Urban and Regional Planners	6	29.42
	**Total**	**60**	
The synchronization with other statutory bodies to meet their requirements.	Architect	13	36.23
	Quantity surveyor	9	26.89
	Builder	24	29.08
	Civil Engineer	8	25.13
	Urban and Regional Planners	6	36.33
	Total	60	
Creating a legal framework for instantaneous and uninterrupted audit and certification of existing buildings.	Architect	13	29.58
	Quantity surveyor	9	29.89
	Builder	24	30.90
	Civil Engineer	8	39.88
	Urban and Regional Planners	6	19.33
	**Total**	**60**

**Table 5:** Kruskal Wallis test statistics on construction experts building control measures exercise.

Building Control Measures	Chi-Square	df	Asmp fig
The handling and examining of building plans for approval.	2.448	4	.654
The assessment of the progress of building work.	1.857	4	.762
The assessment of building completed according to accepted building requirements for giving out certificates of occupancy.	6.254	4	.181
Zero tolerance for illegal, hazardous and violation of statutory requirements related to building	1.019	4	.907
Record keeping in relation to building and related authorizations.	1.524	4	.822
The synchronization with other legal bodies to meet their requirements.	3.820	4	.431
Creating a legal framework for instantaneous and uninterrupted audit and certification of existing buildings.	5.532	4	.237

**Table 6:** Kruskal Wallis result on construction expert mean rank on building control procedures.

Building Control Procedures	Profession	N	Median
The handling and examining of building plans for approval	Architect	13	4
	Quantity surveyor	9	5
	Builder	24	4
	Civil Engineer	8	4
	Urban and Regional Planners	6	4
	**Total**	**60**	**4**
The assessment of the progress of building work.	Architect	13	4
	Quantity surveyor	9	3
	Builder	24	4
	Civil Engineer	8	4
	Urban and Regional Planners	6	3
	**Total**	**60**	**4**
The assessment of building completed according to accepted building requirements for giving out a certificate of occupancy.	Architect	13	3
	Quantity surveyor	9	4
	Builder	24	3
	Civil Engineer	8	3
	Urban and Regional Planners	6	3
	**Total**	**60**	**3**
Zero tolerance for illegal, hazardous and violation of legal requirements related to building	Architect	13	3
	Quantity surveyor	9	3
	Builder	24	3
	Civil Engineer	8	3
	Urban and Regional Planners	6	3
	**Total**	**60**	**3**
Record keeping in relation to building and related authorization.	Architect	13	3
	Quantity surveyor	9	3
	Builder	24	3
	Civil Engineer	8	3
	Urban and Regional Planners	6	3
	**Total**	**60**	**3**
The synchronization with other legal bodies to meet their requirements.	Architect	13	2
	Quantity surveyor	9	2
	Builder	24	2
	Civil Engineer	8	1.5
	Urban and Regional Planners	6	2
	Total	60	**2**
Creating a legal framework for instantaneous and uninterrupted audit and certification of existing buildings.	Architect	13	2
	Quantity surveyor	9	2
	Builder	24	2
	Civil Engineer	8	3
	Urban and Regional Planners	6	1
	**Total**	**60**	**2**

**Table 7:** Ranks of public and private professionals on building control procedures barriers.

	Institution	N	Mean Rank	Sum of Ranks
Barriers to Building Control Measures	private	37	29.19	1080.00
	public	23	32.61	750.00
	**Total**	**60**

**Table 8:** Mann-Whitney U test statistics.

	Barriers to Building Control Measures
Mann-Whitney U	377.000
Wilcoxon W	1080.000
Z	−.740
Asymp. Sig. (2-tailed)	.459

**Table 9:** Barriers to building control procedures median score of public and private construction professionals.

Institution	N	Media
Private	37	40.00
Public	23	41.00
**Total**	**60**	**40.50**

## 2 Experimental design, materials and method

### 2.1 Data collection

A well-structured questionnaire was used to generate the data by targeting a pool of registered construction experts operating in the study area. Random sampling technique was used in selecting the sample size. The contents (variables) of the questionnaire was sourced from the review of literatures and is designed to capture the datasets. A similar method of data exploration includes [10], [11].

### 2.2 Data analysis

The data gathered was analyzed using percentages, mean item score and descriptive statistics as indicated in Table 1, Table 2, Table 3. The data was also analyzed with inferential statistical tool which in Kruskal-Wallis and Mann-Whitney U. Kruskal-Wallis statistical tool was used to test for a significant difference on the perceptions of the construction experts on performance of building control measures in the study area (Table 4, Table 5, Table 6), while Mann-Whiney U was used to test for significant difference between the construction professionals in the public and private group on the barriers militating against building control measures practice (Table 7, Table 8, Table 9). A similar method of data analysis includes [12], [13].
